# A Fully Human Inhibitory Monoclonal Antibody to the Wnt Receptor RYK

**DOI:** 10.1371/journal.pone.0075447

**Published:** 2013-09-18

**Authors:** Michael M. Halford, Maria L. Macheda, Clare L. Parish, Elena A. Takano, Stephen Fox, Daniel Layton, Edouard Nice, Steven A. Stacker

**Affiliations:** 1 Tumour Angiogenesis Program, Peter MacCallum Cancer Centre, East Melbourne, Victoria, Australia; 2 Angiogenesis Laboratory, Ludwig Institute for Cancer Research, Royal Melbourne Hospital, Parkville, Victoria, Australia; 3 Florey Neuroscience Institutes, Melbourne Brain Centre, The University of Melbourne, Parkville, Victoria, Australia; 4 Monash Antibody Technologies Facility, Monash University, Clayton, Victoria, Australia; 5 Sir Peter MacCallum Department of Oncology, The University of Melbourne, Parkville, Victoria, Australia; National Cancer Institute, NIH, United States of America

## Abstract

RYK is an unusual member of the receptor tyrosine kinase (RTK) family that is classified as a putative pseudokinase. RYK regulates fundamental biological processes including cell differentiation, migration and target selection, axon outgrowth and pathfinding by transducing signals across the plasma membrane in response to the high affinity binding of Wnt family ligands to its extracellular Wnt inhibitory factor (WIF) domain. Here we report the generation and initial characterization of a fully human inhibitory monoclonal antibody to the human RYK WIF domain. From a naïve human single chain fragment variable (scFv) phage display library, we identified anti-RYK WIF domain–specific scFvs then screened for those that could compete with Wnt3a for binding. Production of a fully human IgG_1κ_ from an inhibitory scFv yielded a monoclonal antibody that inhibits Wnt5a-responsive RYK function in a neurite outgrowth assay. This antibody will have immediate applications for modulating RYK function in a range of settings including development and adult homeostasis, with significant potential for therapeutic use in human pathologies.

## Introduction

The RTK family regulates a broad spectrum of fundamental metazoan cell behaviors including proliferation, differentiation, metabolism, migration and patterning. Topologically, RTKs are type I transmembrane proteins with an extracellular ligand-binding region, a single-pass hydrophobic transmembrane helix and an intracellular region that contains a protein tyrosine kinase (PTK) domain flanked by additional regulatory sequences. Specific domain combinations in the extracellular region of human RTKs define 20 subfamilies, each characterized by the ability to transduce signals in response to the binding of members of a structurally related group of protein ligands [1]. Intensive study of RTKs has in recent years uncovered surprising diversity in their interactions with other regulatory proteins. For example, interactions with co-receptors (e.g. VEGFR-2 with NRP-1 [2]) and/or activation by ligands previously thought to be recognized exclusively by different receptor classes (e.g. Ror2 by Wnt5a [3]) has enriched our understanding of molecular interactions involving RTKs.

RYK is in many respects an idiosyncratic member of the RTK family [4]. The extracellular region of RYK contains a WIF domain [5] that was originally identified and characterized in the context of the secreted WIF1 protein [6]. The WIF domain functions to sequester vertebrate Wnts or *Drosophila* Hedgehog when present in mammalian WIF1 orthologs [6], [7] or *Drosophila* Shifted [8], [9], respectively. By virtue of its extracellular WIF domain, RYK functions as a cell surface receptor or co-receptor for Wnts. Upon Wnt binding, RYK participates in the activation of β-catenin–dependent [10], [11], [12], [13], [14] or –independent [15], [16], [17], [18], [19], [20], [21], [22], [23] signaling pathways. RYK belongs to a small but biologically significant group characterized by an apparently catalytically inactive PTK domain with atypical variation at one or more normally conserved residues believed to be essential for γ-phosphoryl transfer from ATP to an acceptor tyrosine residue (predicted pseudokinases [24]).

Progress in defining the biological roles of RYK has trailed many of the other RTK members, largely due to the properties of Wnt glycolipoprotein ligands and the apparent pseudokinase status of RYK. However, genetic analyses of *RYK* orthologs and paralogs in model organisms have revealed Wnt-responsive regulatory functions in a wide range of developmental and pathological contexts [4]. Thematically, Ryk subfamily members control important aspects of cell polarity [12], [17], cell differentiation [14], [16], [18], [25], [26], cell migration and target site selection [27], [28], [29], [30], [31], [32], [33], convergent extension movements [17], [19], [21], pattern formation [28], [34], [35], [36], skeletal development [23], [37], neurite outgrowth [11], and axon pathfinding and fasciculation [20], [22], [38], [39], [40], [41], [42], [43], [44], [45], [46]. In rat models of spinal cord and peripheral nerve injury, Wnt/Ryk signaling is rapidly induced on axons and mediates a chemorepulsive response that limits regenerative potential [47], [48], [49], [50]. Delivery of neutralizing anti-Ryk polyclonal antibody prevented corticospinal tract axon retraction from an experimental lesion, caused sprouting of axons at and caudal to the lesion, and enhanced functional recovery after injury [48], [50]. Consistent with these findings, ectopic expression of a secreted Wnt antagonist (WIF1 or sFRP2) by stromal cells grafted at the site of a lesion to central branch dorsal column axons after a peripheral conditioning injury enhanced the central regenerative response [49].

Although RYK now has an established role in the transduction of Wnt-initiated signals, the exact mechanisms by which RYK functions at a molecular and cellular level have remained more elusive. Recently, we showed that RYK can signal via activation of the small GTPase RhoA, although the downstream mediators and effectors of this pathway are largely unknown [17]. Targeted inhibition of RYK function with conventional small molecule ATP-competitive PTK inhibitors has not been pursued due to the absence of evidence for intrinsic PTK activity [4]. While many RYK-interactive partners have been identified (see, for example [13], [23], [37]), they have not yet provided suitable targets for antagonizing RYK function. Attempts to generate inhibitory monoclonal antibodies (MAbs) to RYK have been hampered by poor immunogenicity of the receptor extracellular region and a lack of structural information regarding how the receptor interacts with Wnts and/or Wnt co-receptors.

Here we report the successful generation of a specific and fully human MAb directed to the WIF domain of human RYK. The antibody blocks Wnt binding to RYK and inhibits downstream signaling in a Wnt5a-dependent neurite outgrowth assay. This inhibitory antibody has the potential to therapeutically target RYK activity in pathological states, most notably spinal cord and peripheral nerve injury.

## Results

### Conventional approach to generating MAbs to the RYK extracellular region

**Figure 1:**
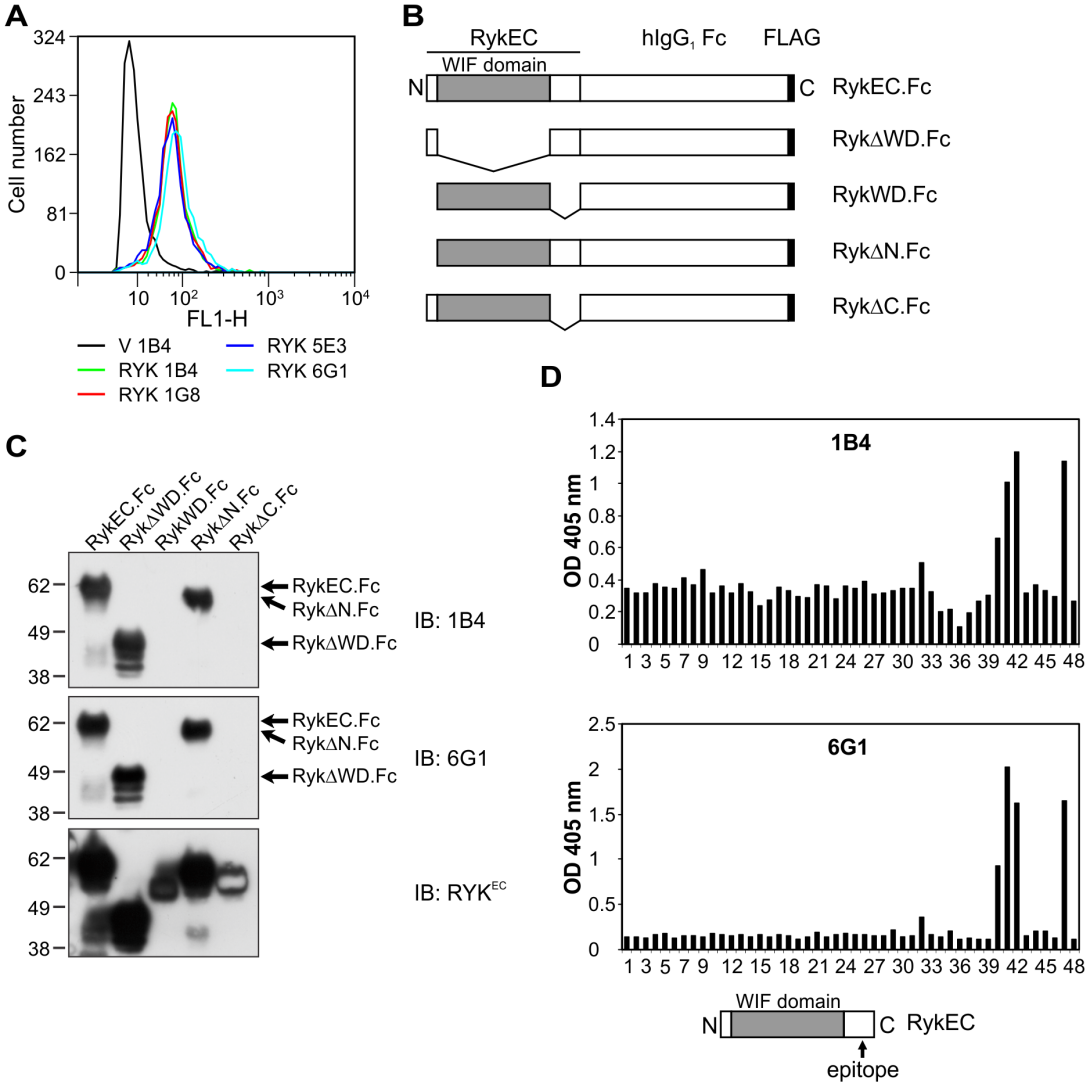
Generation of mouse MAbs to the RYK extracellular region and epitope mapping. (A) Flow cytometry using purified mouse anti-RYK MAbs 1B4, 1G8, 5E3 and 6G1 on 293-EBNA cells stably expressing pVITRO3-mcs (empty vector control; V) or hRYKFCT (RYK). All antibodies detected RYK in hRYKFCT-transfected but not vector-transfected cells. (B) Schematic of the mouse Ryk fusion proteins used in this study. EC, extracellular region; WD, WIF domain. (C) Western blot analysis of purified mouse Ryk fusion proteins using mouse anti-RYK MAbs 1B4 and 6G1. The pattern of binding was the same for both antibodies. The presence of all the fusion proteins was confirmed by stripping the membrane and reprobing with rabbit anti-Ryk^EC^ polyclonal antibody. Molecular mass standards are shown at left in kDa. IB, immunoblot. (D) ELISA results using mouse anti-RYK MAbs 1B4 and 6G1 on an immobilized peptide library of the entire human RYK extracellular region. Peptides 3 to 37: RYK WIF domain; peptide 47: FLAG epitope (incubated with mouse anti-FLAG M2 MAb; positive control); well 48: empty (negative control). The MAbs were used at 2 µg/mL. All antibodies bound to the same epitope, in peptides 40−42. The location of the epitope is shown schematically (bottom). Epitopes for the 1G8 and 5E3 antibodies were identical (data not shown). OD, optical density.

Previously, we generated MAbs to RYK using a soluble version of the entire human RYK extracellular region (H-RYK-FLAG expressed in a mammalian system) as the immunogen ([51]; see Tables S1 and S2 for information regarding antibodies and fusion proteins, respectively, used in this study). However, they were all of the IgM isotype, indicating a lack of *in vivo* B cell class switching and affinity maturation typically seen with antigens that poorly stimulate an immune response. We subsequently performed fusions of mouse splenocytes with myeloma cells after immunization with different recombinant versions of the human RYK extracellular region. Mice immunized with H-RYK-FLAG resulted in several MAbs of the IgG isotype reactive with RYK, named 1B4, 1G8, 5E3 and 6G1. These MAbs were able to detect RYK on cells expressing full-length human RYK (hRYKFCT) by flow cytometry (Fig. 1A).

### Mouse MAbs to RYK bind outside the WIF domain

To map the epitopes recognized by the mouse anti-RYK MAbs, purified mouse Ryk extracellular region-Fc fusion proteins (Fig. 1B; Table S2) were analyzed by Western blotting. All four mouse MAbs recognized the wild-type mouse Ryk extracellular region fusion (RykEC.Fc) and Ryk fusion proteins containing the membrane-proximal region at the carboxyl terminus of the extracellular domain, but not a WIF domain-only fusion (RykWD.Fc) or one with deletion of the region carboxyl-terminal to the WIF domain (RykΔC.Fc; Fig. 1C).

A synthetic peptide library comprising the extracellular region of human RYK was used to further map the epitope(s) recognized by the mouse anti-RYK MAbs. A solid phase binding assay showed that the 1B4 and 6G1 anti-RYK MAbs bound the same cluster of peptides (40–42), which mapped to the region carboxyl-terminal to the WIF domain (Fig. 1D). A second peptide library allowed mapping of the epitope recognized by all four mouse MAbs (1B4, 1G8, 5E3 and 6G1) to the amino acid sequence RTIYD (not shown), which corresponds to residues 212–216 of human RYK. The human RYK WIF domain comprises residues 66–194 (mouse residues 50–178). Therefore, the epitope recognized by all these mouse anti-RYK MAbs maps to an extracellular juxtamembrane region carboxyl-terminal to the WIF domain (Fig. 1D, bottom).

### Proprotein convertase cleavage sites in the RYK WIF domain

**Figure 2:**
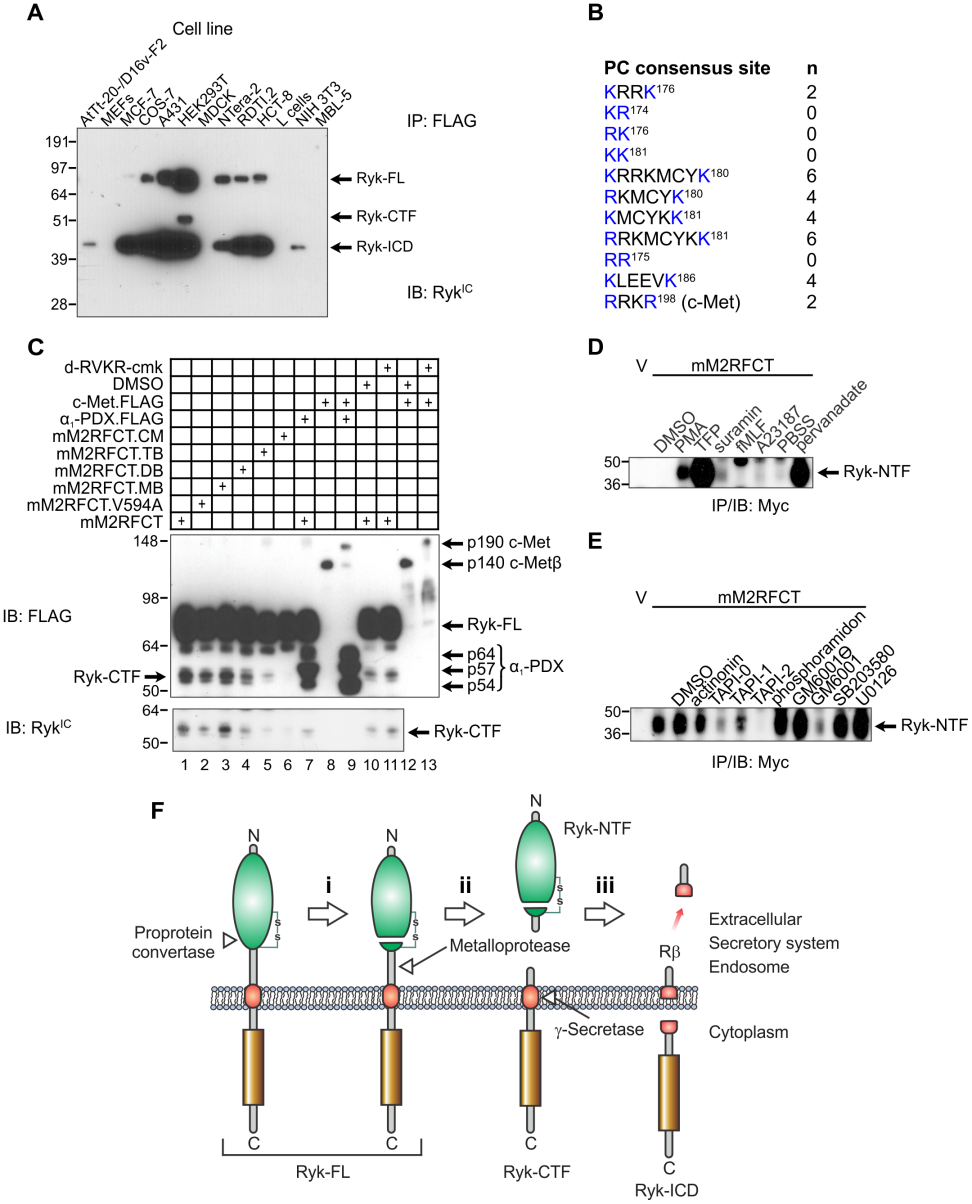
Proteolytic processing of the mouse Ryk extracellular region. (A) Proteolysis of Ryk in mammalian cell lines. Cells were transiently transfected with pcDNA3.mM2RFCT and lysed 48 h later. Anti-FLAG immunoprecipitates (IP) were immunoblotted (IB) with rabbit anti-Ryk^IC^ polyclonal antibody. Molecular mass standards are shown at left in kDa. MEFs, mouse embryonic fibroblasts; RDTI.2, Ryk-deficient large T antigen-immortalized fibroblasts derived from a *Ryk* ^−/−^ embryo. (B) Consensus cleavage sites in the mouse Ryk extracellular region for PC1, PC2, furin, PC4, PC5, PACE4 and PC7 (single-letter amino acid code; basic residues conforming to the consensus in blue; residues numbered according to NCBI Reference Sequence NP_038677.3; (K/R)X_n_(K/R)↓, where X is any residue, n = 0, 2, 4 or 6 and the downward arrow represents cleavage [53]). The furin cleavage site in the mouse c-Met extracellular domain is shown for comparison. (C) COS-7 cells were transiently transfected with plasmids encoding mM2RFCT or the derivatives V594A; K186Q (monobasic, MB); KK181→QQ181 (dibasic, DB); KRRK176→QQQQ176 (tetrabasic, TB); and QQQQ176;QQ181;Q186 (compound mutant; CM). The α_1_-PDX.FLAG protein (p54, p57 and p64 isoforms indicated) was expressed to inhibit endogenous furin. Mouse c-Met.FLAG was expressed as a positive control for inhibition of furin. The location and identity of Ryk-CTF was confirmed using anti-Ryk^IC^ polyclonal antibody (bottom panel). Molecular mass standards are shown at left in kDa. (D) Transiently transfected COS-7 cells were treated with potential activators of receptor shedding for 30 min. Anti-Myc IPs were prepared from the conditioned medium and immunoblotted with an anti-Myc antibody. Molecular mass standards are shown at left in kDa. V, empty vector (pcDNA3)-transfected cells; PBSS, phosphate-buffered saline with calcium and magnesium used as diluent for pervanadate. (E) Transiently transfected COS-7 cells were pre-treated with protease inhibitors, then shedding was activated with TFP (100 µM, 30 min). Anti-Myc IPs were analyzed as in (C). Molecular mass standards are shown at left in kDa. (F) Model for sequential proteolysis of Ryk. The metalloprotease-mediated cleavage in step (ii) has constitutive and inducible components. Green, WIF domain; gold, PTK domain; red, transmembrane helix; Ryk-FL, full-length uncleaved Ryk; Ryk-CTF, Ryk carboxyl-terminal fragment; Ryk-ICD, Ryk intracellular domain fragment; Rβ, predicted membrane-associated peptide analogous to amyloid-β peptide.

We next investigated whether proteolysis of the H-RYK-FLAG immunogen could account for the failure to recover MAbs that recognized the Ryk WIF domain. Full-length mouse Ryk (lacking its signal peptide; Ryk-FL) is cleaved in a stepwise fashion [18]. The first cleavage step, which liberates the soluble WIF domain-containing Ryk-NTF and generates a transmembrane carboxyl-terminal fragment (Ryk-CTF), must occur before the second cleavage step by γ-secretase can take place to produce Ryk-ICD, as is observed with other γ-secretase substrates such as amyloid precursor protein and Notch [52]. Transient transfection of a variety of cell lines with an expression vector encoding a form of mouse Ryk with two amino-terminal Myc epitopes and a **DYKDDDDK**-FHAALGAYV-COOH sequence (mM2RFCT; FLAG epitope bolded) showed that although four cell lines were resistant to transfection, the other nine processed Ryk-FL to Ryk-CTF and Ryk-ICD to variable extents, as revealed by a rabbit anti-Ryk^IC^ polyclonal antibody (Fig. 2A; Table S2). Therefore, constitutive proteolytic processing of Ryk apparently takes place in cell lines with diverse origins.

The RYK extracellular region contains the motif KRRKMCYKKLEEVK in human, mouse, and rat (basic residues bolded; carboxyl-terminal seven residues of the WIF domain underlined), which represents multiple proprotein convertase (PC) consensus cleavage sites (Fig. 2B). Similar basic motifs in secretory proteins are cleaved by PCs during or after transit through the secretory pathway [53]. To determine whether the PC consensus cleavage sites are important for Ryk-CTF generation, COS-7 cells were transiently transfected to express full-length mouse Ryk (mM2RFCT) or derivatives with relevant basic residues (lysine or arginine) substituted with glutamine (Table S2). A∼55 kDa Ryk-CTF was constitutively generated in HEK293T and COS7 cells overexpressing mM2RFCT (Fig. 2A, lane 6; Fig. 2C, lane 1). Constitutive Ryk-CTF levels were substantially reduced by a tetrabasic substitution (Fig. 2C, lane 5) and almost abolished by a compound substitution (Fig. 2C, lane 6). This result suggests that the PC consensus sites, including the KRRK motif, or their positive charge are important for Ryk-CTF generation. PC-specific inhibition using α_1_-PDX or the small molecule decanoyl-RVKR-chloromethylketone (see Table S3 for details of the modulators of proteolysis used in this study) was effective in attenuating proteolytic processing of mouse c-Met (Fig. 2C, lanes 8, 9, 12 and 13), but these agents differentially affected Ryk-CTF generation. α_1_-PDX expression reduced Ryk-CTF generation (Fig. 2C, lane 1 versus 7) while decanoyl-RVKR-chloromethylketone was ineffective (Fig. 2C, lane 10 versus 11). The basis for the differential inhibitory activity of these PC-specific inhibitors towards Ryk-CTF generation is not known. Nevertheless, these findings are consistent with the reported cleavage of Zebrafish (*Danio rerio*) Ryk in the WIF domain somewhere between cysteine residues 155 and 188. The intervening sequence includes the KRRK^186^ motif, and the two resulting chains are linked by a disulfide bond [19].

### Shedding of Ryk-NTF by a metalloprotease

To define the class of protease responsible for simultaneous generation of Ryk-NTF and -CTF, we first tested the responsiveness of mM2RFCT to known activators of receptor shedding [54], [55]. COS-7 cells transiently transfected with a vector encoding mM2RFCT massively upregulated shedding of Ryk-NTF into the medium in response to activation of protein kinase C by phorbol ester, inhibition of protein phosphotyrosyl phosphatases by pervanadate or by the calmodulin inhibitor trifluoroperazine (TFP; Fig. 2D; Table S3). The TFP-induced shedding of Ryk-NTF was inhibited by pretreatment with the metalloprotease-specific inhibitors TAPI-0, TAPI-1 and GM6001, but not the inactive analogue GM6001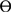, and almost completely by TAPI-2 (Fig. 2E; Table S3). Therefore, cleavage of the extracellular region to generate Ryk-CTF is likely to be mediated by a metalloprotease. Ryk-CTF is embedded in the plasma membrane and exposes a truncated extracellular region which likely contains a more immunogenic epitope than the WIF domain given that all the mouse anti-RYK MAbs generated in this study (and previously [51]) bind to an epitope contained within it (Table S1). Integrating our findings with the data of others [18], [19], we propose a three-stage model for sequential proteolysis of Ryk in which PC-mediated WIF domain cleavage primes the receptor for subsequent scission by a metalloprotease then γ-secretase (Fig. 2F).

### Generation of a human RYK WIF domain–only fusion protein

**Figure 3:**
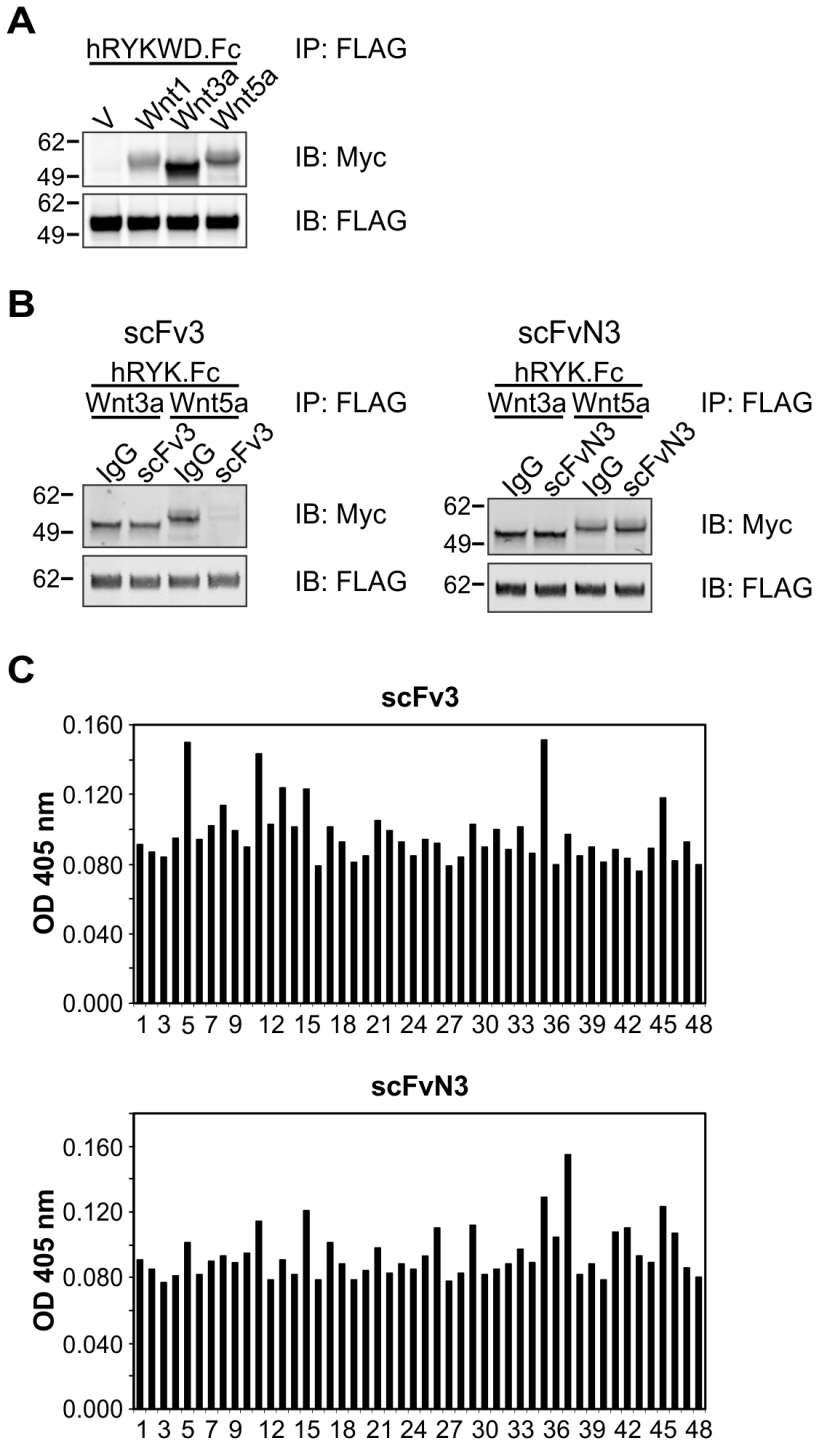
Properties of inhibitory anti-RYK scFvs. (A) Co-IP of Wnt1.Myc5, Wnt3a.Myc5 and Wnt5a.Myc5 from lysates (200 µg) of transiently transfected HEK293T cells with 1 µg purified hRYKWD.Fc protein. Anti-FLAG IPs were immunoblotted (IB) with an anti-Myc antibody to detect co-precipitated Wnt. Molecular mass standards are shown at left in kDa. V, empty vector (pcDNA3)-transfected cells. (B) Anti-FLAG IPs were performed to pull down hRYK.Fc (250 ng), then immunoblotted (IB) as shown. Molecular mass standards are shown at left in kDa. IgG, human IgG control. (C) ELISA analysis of anti-RYK scFv proteins (5 µg/mL) on an immobilized peptide library of the entire human RYK extracellular region (peptides 1 to 46; peptide 47: FLAG epitope; well 48: empty). No linear epitopes were identified. OD, optical density.

To generate MAbs directed specifically to the RYK WIF domain, we expressed a secreted recombinant protein corresponding to the WIF domain of human RYK, lacking the metalloprotease cleavage site, translationally fused to the Fc.FLAG region of human IgG_1_, termed hRYKWD.Fc. When purified from CHO-K1 cell supernatant, the hRYKWD.Fc retained the ability to bind to Wnt proteins as demonstrated by co-immunoprecipitation (co-IP; Fig. 3A).

### Screening a human scFv library for inhibitors of the RYK–Wnt interaction

Given that the RYK WIF domain appeared unlikely to be rendered poorly immunogenic by *in vivo* cleavage, we screened a human naïve scFv phage display library using hRYKWD.Fc to avoid the possible issue of immunological tolerance. The phage display library was screened by enzyme-linked immunosorbent assay (ELISA) for binding to the purified hRYKWD.Fc protein (two rounds). A third round of screening was performed for binding to hRYKWD.Fc in the presence of recombinant Wnt3a protein to select scFv clones that could effectively compete with Wnt3a for binding to the RYK WIF domain. Of the 98 phage clones screened by competitive ELISA in the third round, five were able to compete with Wnt3a for binding to hRYKWD.Fc. The phage DNA of these clones was sequenced and represented two unique scFv sequences, scFv3 and scFvN3.

### Characterization of scFv proteins reactive with RYK

To confirm the activity of the scFv proteins and their capacity to compete with the binding of Wnts, we established an assay for inhibition of Wnt binding to FLAG-tagged hRYK.Fc, a recombinant fusion protein containing the entire human RYK extracellular region. ScFv3 inhibited co-IP of Wnt5a but not Wnt3a with hRYK.Fc, while scFvN3 did not alter the amount of either Wnt3a or Wnt5a bound by hRYK.Fc (Fig. 3B). These results indicate that, at least in this assay, scFv3 but not scFvN3 effectively interferes with RYK binding to Wnt5a.

Mapping of epitopes recognized by the scFv proteins was performed using the same peptide library utilized to map the epitope recognized by the mouse anti-RYK MAbs. Neither of the scFvs bound to consecutive peptides (Fig. 3C), suggesting that the epitope to which each scFv binds is discontinuous and that the scFvs are likely to bind RYK in a conformation-dependent manner.

### Characterization of a fully human inhibitory anti-RYK IgG_1κ_ MAb

**Figure 4:**
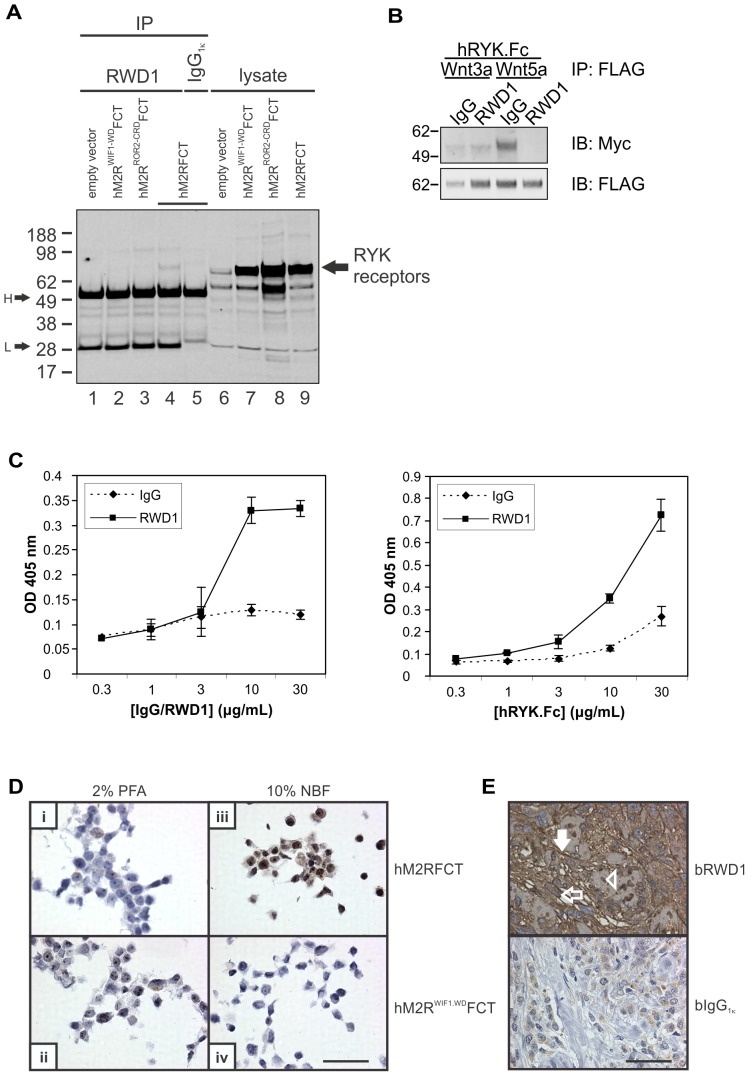
Characterization of the human inhibitory anti-RYK IgG_1κ_ antibody (RWD1). (A) RWD1 IPs from lysates of HEK293T cells transiently transfected with vectors encoding human RYK domain swap derivatives (lanes 1–5) were immunoblotted (IB) with anti-FLAG antibody. Lysate input (lanes 6–9) is shown at right. Molecular mass standards are shown at left in kDa. WIF1-WD, WIF domain from human WIF1; ROR2-CRD, CRD from human ROR2; H, heavy chain; L, light chain. (B) Anti-FLAG IPs were immunoblotted (IB) with an anti-Myc antibody to detect Wnt binding to hRYK.Fc. Molecular mass standards are shown at left in kDa. (C) ELISA analysis of immobilized RWD1 probed with hRYK.Fc. Increased hRYK.Fc binding to the antibody was observed with higher concentrations of either immobilized RWD1 (left panel) or soluble hRYK.Fc (right panel). Results represent the mean±standard deviation of two or three independent experiments. IgG, human IgG; OD, optical density. (D) HEK293T cells transiently transfected with the plasmids indicated (at right) were fixed as shown (above), paraffin-embedded and subjected to IHC using RWD1 biotinylated on *N*-glycan chains. Bar in panel (iv) represents 50 µm. NBF, neutral-buffered formalin; PFA, paraformaldehyde. (E) Example of IHC on a tumor from a formalin-fixed and paraffin-embedded human breast cancer tissue microarray stained with bRWD1 (upper panel) or human MAb isotype control (bIgG_1κ_; lower panel). Open arrow, RYK on a cancer cell; filled arrow, RYK on tumor stroma; arrowhead, RYK-positive cancer cell nucleus; b, biotinylated on *N*-glycan chains. Bar represents 50 µm.

To better evaluate the inhibitory effect of anti-RYK scFvs, scFv3 was grafted onto a human IgG_1κ_ backbone to generate a fully human MAb called RWD1. The binding specificity of purified RWD1 was evaluated in an IP experiment using hM2RFCT and domain swap derivatives in which the human WIF1 or ROR2 Wnt-binding domains (WIF domain or cysteine-rich domain (CRD), respectively) replaced the human RYK WIF domain (Table S2). From lysates of transiently transfected HEK293T cells, RWD1 precipitated hM2RFCT (Fig. 4A, lane 4) but not when the RYK WIF domain had been substituted with that from WIF1 (Fig. 4, lane 2) or the CRD from ROR2 (Fig 4, lane 3). This result demonstrates high binding specificity of RWD1 for its epitope in the RYK WIF domain. Epitope mapping using RWD1 on the RYK extracellular region peptide library confirmed that there was no binding to consecutive peptides (not shown). The ability of RWD1 to interfere with binding of Wnt by RYK was evaluated in a co-IP experiment (Fig. 4B), which confirmed inhibitory activity towards the Wnt5a/RYK interaction but not the Wnt3a/RYK complex. Binding of the antibody to RYK was further tested by ELISA, which showed increased binding of hRYK.Fc ligand to immobilized RWD1 MAb with increasing concentrations of either MAb or ligand (Fig. 4C).

We screened for conditions that would allow us to use RWD1 as a primary immunohistochemical (IHC) reagent to detect RYK expression on human cells and tissues. Labeling of RWD1 was desirable so that problematic anti-human IgG secondary reagents could be omitted. However, RWD1 contains multiple lysine residues in its complementarity-determining regions and the use of activated labeling reagents that target primary amine groups was therefore avoided so that full binding activity could be retained. RWD1 with (polyethylene glycol)_4_-biotin groups covalently linked to its *N*-glycan chains (bRWD1), in combination with neutral-buffered formalin fixation and paraffin embedding, specifically detected hM2RFCT but not hM2R^WIF1-WD^FCT in transiently transfected HEK293T cells (Fig. 4D). Evaluation of bRWD1 versus a similarly biotinylated fully human IgG_1κ_ MAb, which recognizes a hapten not present in mammalian tissues, on a human breast cancer tissue microarray demonstrated expression of RYK on stroma and cancer cells in tumor cores (Fig. 4E).

### Binding affinity of RWD1 MAb for RYK

**Figure 5:**
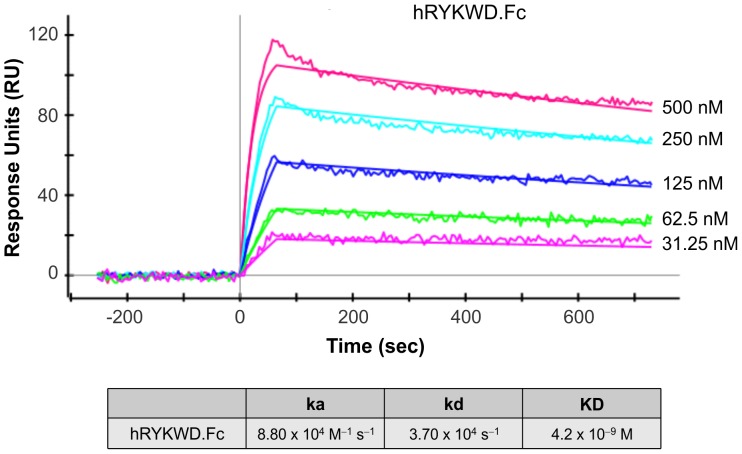
Surface plasmon resonance imaging (SPRi) analysis of the interaction between immobilized RWD1 and hRYKWD.Fc. Experiments were performed using a ProteOn XPR36 SPRi biosensor equipped with a GLC chip bearing immobilized RWD1 as described in Materials and Methods. Five concentrations of ligand (hRYK.Fc; indicated at right) and a buffer blank were analyzed simultaneously. Calculated kinetic constants are shown at bottom.

Surface plasmon resonance imaging (SPRi; [56]) was used to determine the affinity of RWD1 MAb for the human RYK WIF domain. Purified hRYKWD.Fc was used as the specific binding domain to assess MAb binding. Following a double reference background subtraction protocol (see Materials and Methods), data was fitted globally using a model describing a 1∶1 Langmuirian interaction with mass transfer (Fig. 5). These studies revealed an association rate constant (ka) of 8.80×10^4^ M^–1^ sec^–1^ and a dissociation rate constant (kd) of 3.70×10^4^ sec^−1^, giving a calculated dissociation constant (KD) of 4.2×10^–9^ M.

### Inhibition of Wnt signaling and neurite outgrowth by RWD1 MAb

**Figure 6:**
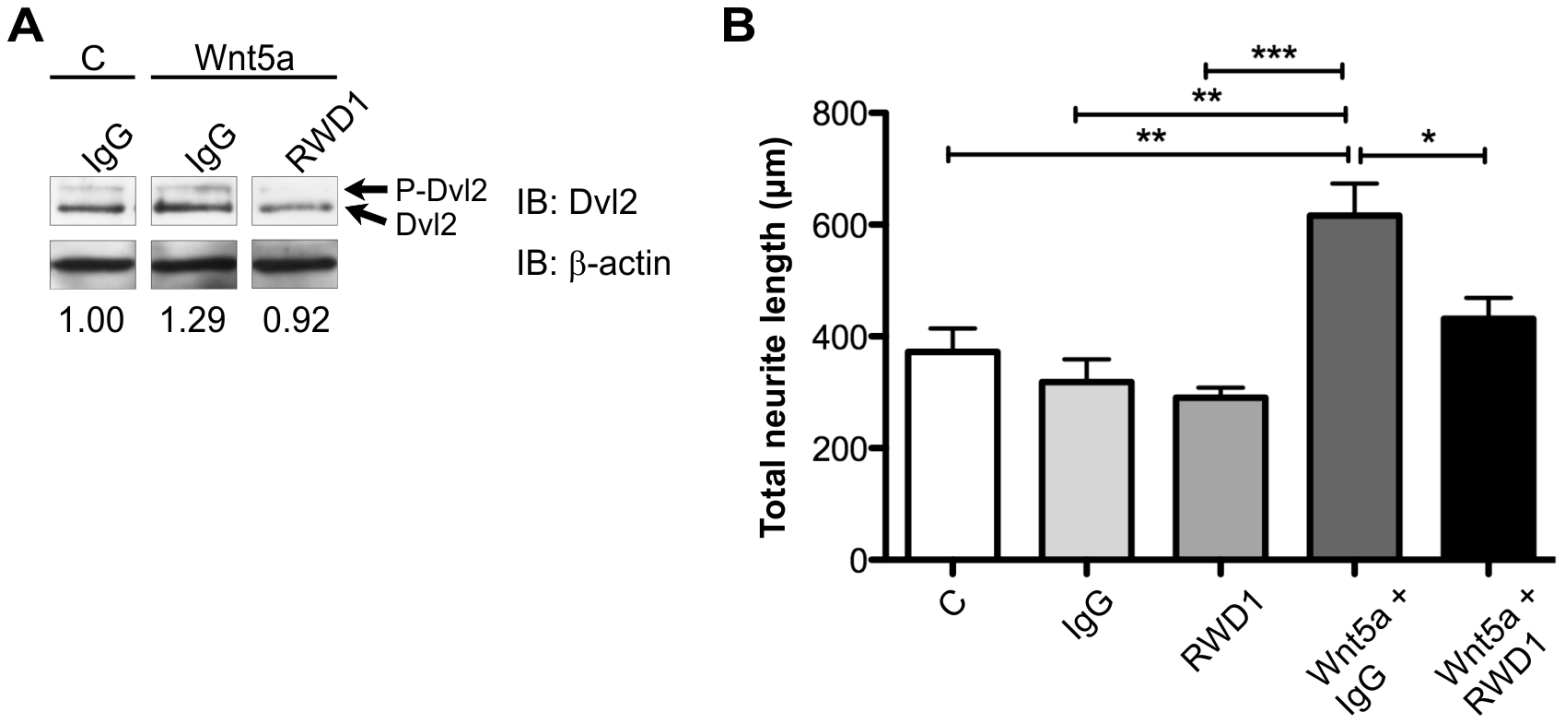
RWD1 inhibits Wnt signaling and Ryk function in neurons. (A) Western blot analysis of lysates from SN4741 cells treated with Wnt5a (300 ng/mL) or vehicle (PBS; C) and with human IgG (50 µg/mL) or RWD1 (50 µg/mL). P-Dvl2, phosphorylated Dvl2. Molecular mass standards are shown at left in kDa. The three lanes were non-consecutive but on the one membrane. The normalized ratio P-Dvl2/total Dvl2 (corrected for β-actin) is shown beneath each lane. (B) Quantification of neurite growth from E15.5 mouse cortical neurons treated with human IgG (50 µg/mL), RWD1 (50 µg/mL), Wnt5a (300 ng/ml) and/or vehicle (PBS; C). Results represent the mean±SEM of four independent experiments. *P<0.05; **P<0.01; ***P<0.001.

The mouse cell line SN4741 [57] was used to confirm that RWD1 can inhibit Wnt-induced signaling. SN4741 cells respond to Wnt5a treatment by phosphorylation of Dishevelled (Dvl) 2 and Dvl3, which are cytoplasmic proteins that transduce both β-catenin–dependent and –independent Wnt signals [58]. Cells treated with Wnt5a showed evidence of Dvl2 activation by phosphorylation (Fig. 6A, lane 1 versus 2) while co-treatment with RWD1 inhibited Wnt5a-induced Dvl2 phosphorylation (Fig. 6A, lane 2 versus 3).

Wnt-activated Ryk signaling stimulates axonal growth during rodent embryonic development [11], [20], [38]. To further examine whether the RWD1 MAb was able to inhibit Ryk function, a neurite outgrowth assay was performed using mouse embryonic day 15.5 cortical neurons. Treatment of neurons with RWD1 alone did not affect neurite outgrowth compared to IgG_1_ control-treated cells (Fig. 6B). Wnt5a treatment significantly increased total neurite length, while co-treatment with RWD1 significantly inhibited Wnt5a-induced neurite outgrowth (Fig. 6B). Together, these results demonstrate that the RWD1 MAb can antagonize Wnt5a/Ryk-induced signaling as assessed by inhibition of Dvl2 phosphorylation and neurite outgrowth.

## Discussion

Aberrant signaling by RTKs is an etiologic factor in cancer and other non-malignant diseases [1], [59], [60]. Neutralizing antibodies against the extracellular region of RTKs or their ligands, ligand traps based on fusions to ligand-binding domains in RTK extracellular regions, and ATP-competitive small molecule PTK inhibitors are currently in clinical use. Such agents are also valuable *in vitro* and *in vivo* probes of RTK function.

We have identified human scFvs from a phage display library that recognize the human RYK WIF domain. One scFv was able to compete with Wnt for binding to the human RYK extracellular region. Epitope mapping showed that this scFv and the corresponding full-length IgG_1κ_ antibody, RWD1, bind to a non-linear epitope of RYK. RWD1 had an affinity (KD) of 4.2×10^−9^ M for hRYKWD.Fc, as determined by SPRi. Significantly, this MAb was able to inhibit Wnt5a-stimulated neurite outgrowth from mouse cortical neurons. Therefore, we have developed a MAb to the human RYK extracellular region that is capable of inhibiting Wnt5a-responsive Ryk function in neuronal cells. We are uncertain why scFv3 competed with Wnt3a for binding hRYKWD.Fc in the phage display screen but then, when purified or grafted onto an IgG_1κ_ backbone to create RWD1, demonstrated inhibitory activity against the Wnt5a/RYK interaction and not Wnt3a/RYK. RWD1 may be a Wnt-specific antagonist of RYK signaling, although we have not yet tested its activity towards the interaction between RYK and ligands other than Wnt3a and Wnt5a. Any such selectivity of RWD1 could be exploited as a research tool and/or in a clinical context, where improved safety and therapeutic index may result from targeting a specific subset of Wnt/RYK complexes. Alternatively, RWD1-mediated competitive inhibition of the binding of specific Wnts by the RYK WIF domain may be sensitive to assay conditions or the post-translational modification status of Wnts and/or the RYK WIF domain.

It has been demonstrated previously that mouse Ryk is proteolytically processed in two steps. The first cleavage event removes the bulk of the extracellular region and primes Ryk-CTF for cleavage in its transmembrane helix by the γ-secretase complex [18]. Consequently, three soluble fragments are produced: an extracellular Ryk-NTF, an intracellular Ryk-ICD and a predicted membrane-associated Rβ peptide [4]. A third site of cleavage in the extracellular domain, between the two conserved cysteine residues of the WIF domain, has been described in zebrafish Ryk [19]. This carboxyl-terminal region of the Ryk WIF domain contains four consecutive basic amino acids conserved in vertebrate RYK proteins (human, mouse, zebrafish and *Xenopus laevis*; KRRK) and in two of the three *Drosophila* RYK subfamily members, Derailed (KRKK) and Doughnut (RRKK). In the current study, we demonstrated the importance of this tetrabasic motif — a likely processing site for PCs — for constitutive shedding of Ryk-NTF. Using a calmodulin inhibitor to upregulate shedding of Ryk-NTF, we have further shown that this second cleavage event carboxyl-terminal to the WIF domain is mediated by a member of the metalloprotease family. We therefore propose a three-stage model for proteolytic processing of Ryk (Fig. 2F). Further experimentation is required to identify the proteases that process the Ryk extracellular region, their exact sites of cleavage (including that for γ-secretase) and their modes of regulation.

Our previous efforts to generate mouse MAbs to the human RYK extracellular domain have resulted in few antibodies that were all of the IgM isotype [51], indicating poor immunogenicity and/or a strong barrier to breaking immunological tolerance. In the current study, we generated four new mouse MAbs to the human RYK extracellular region (1B4, 1G8, 5E3 and 6G1). These four mouse MAbs bind to the same epitope, RTIYD, located at the carboxyl-terminal end of the extracellular region. Our attempts to generate antibodies to the RYK WIF domain alone in mice and rabbits failed to produce any antibodies to RYK that were detectable in the serum (data not shown). This led us to initiate the screen of a human phage display scFv library described here, which resulted in the successful identification of anti-RYK scFvs, one of which (scFv3) inhibited RYK binding to Wnt5a.

Existing data provide evidence for a potent effect of Wnt/Ryk-mediated signaling on inhibition of axon growth and recovery after nerve injury [48], [50]. Consequently, a fully human inhibitory anti-RYK MAb could be used therapeutically in the context of human spinal cord and/or peripheral nerve injury. In addition, such an antibody may have other therapeutic applications, most obviously in the treatment of Wnt-dependent cancers. RYK overexpression has been observed in human ovarian cancer and was correlated with decreased overall survival of patients [61], [62]. Our IHC data, generated using RWD1 biotinylated specifically on *N*-glycan chains, demonstrates RYK expression on the stroma and cancer cells of human breast tumors. Some ovarian- and breast cancer-derived cell lines are dependent on autocrine Wnts [63], [64], suggesting that an inhibitory anti-RYK MAb may benefit a subset of patients with such tumors.

RWD1 has significant advantages over existing antibodies to RYK. It is a human MAb capable of inhibiting RYK signaling in response to at least one Wnt ligand, and the cellular consequences of RWD1 activity represent antagonism of the known functions of the Ryk receptor in a neurite outgrowth assay. RWD1 could therefore provide significant benefit to patients with nerve injury and/or a subset of cancers. Preclinical characterization and validation of the effects of RWD1 in rodent models of spinal cord injury and cancer will be necessary. These studies will provide further insight into the function of RYK in disease states and extend our knowledge of RYK function in normal physiology.

## Materials and Methods

### Ethics statement

Mice were maintained in strict accordance with the Australian National Health and Medical Research Council's “Code of Practice for the Care and Use of Animals for Scientific Purposes, 2004”. No experimental procedures were performed on live animals. Protocols for the use of mice were approved by the Animal Ethics Committees (AECs) at the Ludwig Institute for Cancer Research, Parkville (AEC 25/03) and the Howard Florey Institute (AEC 10-010). Mice were sacrificed by cervical dislocation.

### Cell lines and drug treatment

HEK293T, CHO-K1, AtT-20/D16v-F2, MCF-7, A431, NTera-2, MDCK, HCT-8, L cells and NIH 3T3 cells were from the ATCC. Mouse embryonic fibroblasts (MEFs) and Ryk-deficient large T antigen-immortalized (RDTI) derivatives were derived from wild-type or *Ryk^–/–^* mouse embryos, respectively. MBL-5 cells were provided by Dianne Grail [65]; COS-7 cells were from Brian Seed (Harvard University) and 293-EBNA cells were from Life Technologies. Cells were maintained in DMEM or RPMI 1640 (Life Technologies) supplemented with 10% heat-inactivated FBS. H-RYK-FLAG/CHO cells were maintained in glutamine-free GMEM (SAFC) supplemented with 10% heat-inactivated and dialyzed FBS, GS supplement (SAFC) and 25 µM methionine sulfoximine (glutamine synthase inhibitor; Sigma-Aldrich). Hybridomas were maintained in Hybridoma-SFM (Life Technologies) with 10% heat-inactivated FBS. SN4741 cells [57] were maintained in DMEM with 10% FBS, 50 U/ml penicillin/streptomycin and 0.6% glucose. All cell lines were incubated at 37°C in 5% or 10% CO_2_. Pervanadate solutions were freshly prepared as described [66].

### Plasmids

The human RYK extracellular region constructs pApex-3.hRYK.Fc.FLAG and pApex-3.hRYKWD.Fc.FLAG were described previously [38]. Human RYK extracellular region construct H-RYK-FLAG, encoding the human RYK extracellular domain fused to a FLAG epitope tag at the carboxyl terminus, was cloned into the pEE6-CMV vector encoding the glutamine synthase cDNA, thus producing pEE6/H-RYK-FLAG. Full-length human RYK with an amino-terminal 2× Myc epitope tag (pcDNA3.Myc2.hRYK) was described previously [17]. DNA encoding full-length human RYK, with a mouse IL-3 signal peptide and a FLAG epitope tag inserted near the carboxyl terminus (between residues 598 and 599; NCBI Reference Sequence NP_002949.2), was subcloned into pVITRO3-mcs (InvivoGen) to produce pVITRO3.hRYKFCT.

A double-tagged version of the mouse *Ryk* cDNA was created from pcDNA3.Myc2.Ryk [37], by inserting in-frame a sequence encoding a FLAG epitope tag between codons 585 and 586 (NCBI Reference Sequence NP_038677.3), to produce Myc2.Ryk.FLAG.CT (M2RFCT). Mutants of pcDNA3.M2RFCT were produced using the QuikChange Site-Directed Mutagenesis Kit (Stratagene): K186Q (monobasic mutant, MB), KK181 to QQ181 (dibasic mutant, DB), KRRK176 to QQQQ176 (tetrabasic mutant, TB) and KRRK176;KK181;K186 to QQQQ176;QQ181;Q186 (compound mutant, CM). Mouse Ryk extracellular domain constructs pApex-3.RykEC.Fc.FLAG, pApex-3.RykΔWD.Fc.FLAG, pApex-3.RykWD.Fc.FLAG, pApex-3.RykΔN.Fc.FLAG and pApex-3.RykΔC.Fc.FLAG were described previously [17].

A FLAG-tagged α_1_-antitrypsin Portland (α_1_-PDX) cDNA [67] was kindly provided by Dr. Gary Thomas and was subcloned into pcDNA3. The mouse *c-Met* cDNA was amplified by RT-PCR using mouse post-natal day 1 head cDNA template, with a reverse PCR primer that encoded a carboxyl-terminal FLAG epitope tag, and was cloned into the *Hin*d III and *Not* I sites of pcDNA3 to produce pcDNA3.c-Met.FLAG. Mouse Wnt constructs pcDNA3.Wnt1.Myc5, pcDNA3.Wnt3a.Myc5 and pcDNA3.Wnt5a.Myc5 were described previously [17].

The sequence encoding the WIF domain in pcDNA3.hM2RFCT (amino acid residues 65–195; NCBI Reference Sequence NP_001005861.1) was replaced with DNA encoding the WIF domain of human WIF1 (amino acid residues 35–178; NCBI Reference Sequence NP_009122.2) or the CRD of human ROR2 (amino acid residues 170–309; NCBI Reference Sequence NP_004551.2) using sequence- and ligation-independent cloning [68] to produce hM2R^WIF1-WD^FCT and hM2R^ROR2-CRD^FCT, respectively.

### Protein production and purification

Stable cell lines hRYK.Fc/CHO and hRYKWD.Fc/CHO were generated as described previously [38]. CHO-K1 cells were transfected with pEE6/H-RYK-FLAG using FuGENE 6 (Roche Diagnostics) and selection was applied after 24 h using medium containing 25 µM methionine sulfoximine. H-RYK-FLAG/CHO, hRYK.Fc/CHO and H-RYK-FLAG/CHO cells were grown on pleated-surface roller bottles (BD Biosciences) and incubated at 37°C in a normal air atmosphere. Conditioned medium was collected after five days, at which time new medium was added and collected again after a further three days. hRYK.Fc and hRYKWD.Fc proteins were produced as described [38]. To produce RykEC.Fc.FLAG, RykΔWD.Fc.FLAG, RykWD.Fc.FLAG, RykΔN.Fc.FLAG and RykΔC.Fc.FLAG proteins, 293-EBNA cells were transiently transfected with the respective expression vector and conditioned medium was collected after five days. Conditioned medium was filtered using 0.22 µm filters (Millipore) and secreted protein was purified using anti-FLAG M2 affinity gel (Sigma-Aldrich) as described previously [69].

### RYK MAb production and purification

Two BALB/c mice were administered H-RYK-FLAG protein three times (WEHI Monoclonal Antibody Facility, Bundoora, Australia). Mouse sera were screened for antibody production by flow cytometry using HEK293T cells transiently transfected with pcDNA3.Myc2.hRYK using FuGENE 6. Anti-Myc 9E10 antibody (WEHI Monoclonal Antibody Facility) was used as a positive control for cell transfection, while propidium iodide (Sigma-Aldrich) was used to identify dead cells. Mice shown to have anti-RYK antibodies were boosted once more with H-RYK-FLAG, then four days later the spleens of the mice were isolated and splenocytes were fused to myeloma cells to produce hybridomas (WEHI Monoclonal Antibody Facility). Hybridoma supernatants were screened by flow cytometry as described above.

To produce and purify mouse anti-RYK MAbs, hybridoma clones were grown in pleated-surface roller bottles in Hybridoma-SFM supplemented with 1% heat-inactivated FBS at 37°C in a normal air atmosphere for nine days. Conditioned medium was passed through 0.22 µm filters, then antibodies were isolated using Protein A sepharose (Fast Flow 4; GE), followed by IgG elution with 50 mM glycine, pH 3.0, into tubes containing 0.2 elution volumes of 1 M Tris-HCl, pH 8.0. Antibody fractions were exchanged into phosphate-buffered saline (PBS) and concentrated using Amicon Ultra units (Millipore).

### RYK polyclonal antibodies used for Western blot analysis

Two polyclonal antibodies to RYK were generated in rabbits. *E. coli* BL21-SI (Life Technologies) was transformed with the pET.GEX.mRykIC vector [70] to express a glutathione *S*-transferase (GST) translational fusion to the N-terminus of the entire intracellular region of mouse Ryk. Glutathione-sepharose affinity-purified GST.mRykIC was used to generate rabbit anti-Ryk^IC^ antiserum. Whole rabbit serum from immunized animals was heat-inactivated, 0.22 µm-filtered and depleted of antibodies to GST by incubation with GST-coupled AffiGel 15 matrix (Bio-Rad Laboratories). Anti-Ryk^IC^ antibodies were affinity-purified using GST.mRykICD-coupled AffiGel 15, eluted [71] and buffer-exchanged into PBS by diafiltration. Purified hRYK.Fc was similarly used to generate rabbit anti-RYK^EC^ antiserum. Anti-RYK^EC^ antibodies were affinity purified from anti-Fc.FLAG–depleted whole serum using immobilized hRYK.Fc as described for anti-Ryk^IC^.

### Mapping of MAb epitopes by Western blot and peptide library ELISA

To begin epitope mapping of anti-RYK monoclonal antibodies, RykEC.Fc.FLAG fusions (45 ng) were separated on 4–12% reducing NuPAGE Novex Bis-Tris gels (Life Technologies). Western blotting was performed as described previously [41] using a 20-fold dilution of hybridoma supernatants containing mouse anti-RYK MAb.

To finely map the binding epitope of anti-RYK MAbs, two PepSet peptide libraries of the human RYK extracellular region were created (Mimotopes, Clayton, Australia). The first PepSet library comprised the entire extracellular region, consisting of 46 peptides of 16 amino acid residues each with a four residue offset. The second PepSet library comprised the region to which anti-RYK MAbs bound in the first PepSet library (residues 199–226 of human RYK), consisting of 19 peptides of 10 residues each with a one residue offset. Peptides from both PepSet libraries had an amino-terminal biotin group followed by a four amino acid residue spacer, allowing the binding of peptides to streptavidin-coated 96-well plates (Mimotopes). ELISAs were performed using purified MAbs at 2 µg/mL or purified scFv proteins at 5 µg/mL. Secondary antibodies were goat anti-mouse IgG-HRP (Bio-Rad Laboratories) and goat anti-human IgG-HRP (Life Technologies).

### Analysis of RYK cleavage events

Mouse Ryk constructs encoding substitutions in the PC consensus sites were introduced into COS-7 cells by transient transfection using FuGENE 6. Medium was changed 24 h later and cells were treated with the furin inhibitor decanoyl-RVKR-chloromethylketone (Bachem) or an equal volume of DMSO for 12 h before harvesting. Forty-eight h post-transfection, cells were washed twice with cold PBS before lysis with 1 mL of cold lysis buffer (50 mM Tris pH 7.5, 150 mM NaCl, 1% Triton X-100) supplemented with 1 mM Na_3_VO_4_ and 1× Complete protease inhibitor cocktail (Roche Diagnostics) for 30 min. Insoluble material was removed by centrifugation at 16,000 *g*, 15 min, 4 °C and the supernatant was used for analysis. Protein concentrations were determined using the BCA Protein Assay (Thermo Scientific) and IP was performed from equal amounts of lysate protein using anti-FLAG M2 affinity gel. IPs were washed three times with lysis buffer and bound proteins were eluted in SDS-PAGE sample buffer. Anti-FLAG M2-HRP (Sigma-Aldrich) was utilized in Western blotting to detect FLAG-tagged proteins.

For analysis of Ryk-NTF shedding, COS-7 cells were transiently transfected with pcDNA3.M2RFCT using FuGENE 6. Cells were treated 36 h post-transfection with protease inhibitors in fresh medium for 12 h. Trifluoroperazine (100 µM; Sigma) was added for the final 30 min of inhibitor treatment to stimulate shedding. Conditioned medium was collected and clarified by centrifugation at 10,000 *g*, adjusted to 0.2% Triton X-100, 0.05% NaN_3_, 100 mM HEPES pH 7.4, and concentrated 10-fold by ultrafiltration using Centricon YM-10 filters (Millipore). Concentrated conditioned medium was used for IP experiments with anti-Myc MAb antibody 9E10-conjugated sepharose as described above.

### Phage display antibody screening

A phage display antibody library screen was performed using a combination of a direct binding assay for purified hRYKWD.Fc and a competitive ELISA (CD BioSciences Inc., NY, USA). The screening protein, purified hRYKWD.Fc, was used on a human scFv naïve phage display library in three rounds of screening. The final (third) round of screening was performed using recombinant mouse Wnt3a protein (R&D Systems) in competitive ELISA. The cDNA of phage clones was sequenced and the two phage clones able to compete with Wnt3a for binding to hRYKWD.Fc (scFv3 and scFvN3) were codon-optimized to remove stop codons contained in the scFv sequence, thus allowing expression of the scFvs in bacteria (CD BioSciences Inc.). Small-scale purification of both 6× His-tagged scFv proteins was performed on a nickel sepharose high-performance column. Eluted material was buffer-exchanged into 50 mM Tris, 50 mM NaCl, 0.1 mM EDTA, 10% glycerol.

### Anti-RYK scFv and IgG inhibition of RYK binding to Wnts *in vitro*

HEK293T cells were transiently transfected with pcDNA3.Wnt3a.Myc5 or pcDNA3.Wnt5a.Myc5. Cells were lysed 24 h post-transfection as described above. Pre-incubation of 250 ng purified hRYK.Fc with 20 µg scFv or IgG was performed at 4°C for 1 h, at which time lysate from Wnt3a.Myc5- or Wnt5a.Myc5-transfected cells (10–20 µg) and anti-FLAG M2 affinity gel were added and incubated at 4°C for 1 h. IPs were washed twice with each of wash 1 (150 mM NaCl, 50 mM Tris, 0.1% Triton X-100, pH 7.5), wash 2 (500 mM NaCl) and wash 3 (50 mM Tris, pH 7.5). IP proteins were eluted in SDS-PAGE sample buffer for Western blotting using anti-FLAG M2 (Sigma-Aldrich) conjugated to IRDye 800CW (LI-COR Biosciences) to detect FLAG-tagged hRYK.Fc, or anti-Myc tag rabbit polyclonal antibody (Abcam) followed by goat anti-rabbit IgG IRDye 680 (LI-COR Biosciences) secondary antibody to detect Myc-tagged Wnts.

To assess inhibition of Wnt signaling by RWD1 antibody in SN4741 cells, 12-well plates were seeded with 10^5^ cells per well and grown overnight in the absence of serum. Cells were pre-incubated for 45 min in the same medium with human IgG (R&D Systems; 50 µg/mL) or RWD1 MAb (50 µg/mL) then stimulated for 2 h with Wnt5a (R&D Systems; 300 ng/mL). Preparation of lysates and immunoblotting were carried out as previously described [38].

### Production of IgG_1κ_ from scFv3

The cDNAs of heavy chain and light chain variable regions from scFv3 were subcloned into two separate vectors, one encoding human κ light chain (pSTDLH3.RYK) and the other human γ1 heavy chain (pSTDHH3.RYK; CD BioSciences Inc.). The vectors encoding heavy and light chains were transiently transfected into FreeStyle 293-F cells using FreeStyle MAX Expression System (Life Technologies). The antibody was purified from conditioned medium six days post-transfection using protein A chromatography (GE) and buffer-exchanged into PBS.

Large-scale purification of RWD1 antibody was performed using a stable cell line. CHO-K1 cells were co-transfected with pSTDHH3.RYK and pSTDLH3.RYK (6∶4 ratio) using Lipofectamine 2000 (Life Technologies) and selection was applied after 24 h by adding 300 µg/mL zeocin (Life Technologies) to the medium. Colonies were picked after 7–9 days. An RWD1/CHO stable cell line was seeded into a medium FiberCell cartridge, 20 kDa (FiberCell Systems), using DMEM+10% FBS+200 µg/mL zeocin, and then maintained in DMEM+10% CDM-HD serum replacement (FiberCell Systems)+150 µg/mL zeocin. Extracapillary space medium was collected every 2–3 days. Antibody purification was performed as described above and RWD1 was buffer-exchanged into citrate buffer (25 mM trisodium citrate, 150 mM NaCl, pH 6.5).

### Biotinylation of RWD1 on *N*-glycan chains

Three mg of purified RWD1 in coupling buffer (100 mM sodium acetate, 150 mM NaCl, pH 5.5 at 23°C) was treated with 20 mM sodium *meta*-periodate (Thermo Fisher Scientific) for 30 min at room temperature to oxidize *cis*-diols on *N*-glycan chains to aldehyde groups. Sodium *meta*-periodate was subsequently removed by diafiltration against coupling buffer and aldehyde groups were targeted for biotinylation using 5 mM alkoxyamine-PEG_4_-biotin and 100 mM aniline catalyst (both from Thermo Fisher Scientific) at room temperature for 18 h with end-over-end mixing. Diafiltration was performed to remove biotinylation reagents and exchange biotinylated RWD1 (bRWD1) into citrate buffer. The concentration of bRWD1 was determined by BCA assay (Thermo Fisher Scientific). A Fluorescence Biotin Quantitation Kit (Thermo Fisher Scientific) was used to determine the stoichiometry of biotinylation, (3.3 mol biotin/mol RWD1). *Cis*-diols on a fully human anti-hapten IgG_1κ_ MAb (Eureka Therapeutics) were oxidized as above then biotinylated in 50 mM sodium phosphate, 150 mM NaCl, pH 7.0 at 23°C and exchanged into PBS for use as an isotype control (3.1 mol biotin/mol MAb) for IHC.

### IP to evaluate RWD1 binding specificity

HEK293T cells in 15 cm dishes were cotransfected with RYK-encoding plasmids and pEGFP-N2 (Clontech) in a 5∶1 mass ratio using polyethylenimine “MAX” (Polysciences, Inc.) as described by Kuroda *et al.* [72], with the exception that the medium used throughout was DMEM, 10% FBS, 2 mM GlutaMAX I, 1 mM sodium pyruvate, 100 U/mL penicillin and 100 µg/mL streptomycin. At 48 h post-transfection, cells were washed with cold DPBS and lysed with cold Buffer 12 (25 mM Tris base, 1% (v/v) glycerol, 2.5 mM CaCl_2_, 2.5 mM MgCl_2_, final ionic strength adjusted to 0.1 with NaCl, 1% Igepal CA 630 and 1× Complete Protease Inhibitor Cocktail (EDTA-free, Roche), pH 8.4 at 4°C). Lysate was cleared at 17,000 *g*, 10 min, 4°C and the supernatant subjected to ultracentrifugation at 100,000 *g*, 1 h, 4°C. BCA assay was performed to quantify protein concentration. Immunoprecipitation (5 µg RWD1 or fully-human IgG_1κ_ isotype control) was performed from equal amounts of lysate protein at equal concentrations for 15 h at 4°C with end-over-end rotation. Immune complexes were captured by incubation with BSA-blocked protein A-sepharose (GE) for a further 1 h. Beads were washed three times for 5 min each (with end-over-end rotation) in cold Buffer 12. Immune complexes were eluted at 70°C for 10 min in 1× Bolt LDS Sample Buffer (Life Technologies) containing 25 mM TCEP. Eluted proteins were fractionated on 4–12% Bolt Bis-Tris Plus gels in 1× MES buffer (Life Technologies) and electroblotted to a nitrocellulose membrane. The membrane was blocked, incubated with anti-FLAG (clone M2, Sigma Aldrich) conjugated to IRDye 800 (LI-COR Biosciences) then washed and imaged using an Odyssey Infrared Imager (LI-COR Biosciences).

### Production of cell blocks

HEK293T cells (at 48 h post-transfection) were washed with DPBS then detached by incubation at 37°C in citric saline (15 mM trisodium citrate, 135 mM KCl) for 5–10 min. Cells were collected by centrifugation (150 *g*, 5 min, room temperature), washed again with DPBS and resuspended in neutral-buffered formalin or cold 2% paraformaldehyde in DPBS. Cells were fixed for 30 min at 4°C or 2 h at room temperature, respectively. After washing with DPBS, fixed cells were resuspended in liquid 4% low melting point agarose in DPBS and molds (inverted 1.5 mL tube lids) were filled then placed on ice to harden. Cell blocks were post-fixed overnight in the respective fixative before being embedded in paraffin and sectioned for immunohistochemistry.

### Immunohistochemistry

Three µm-thick sections were dewaxed and hydrated through a descending ethanol series and equilibrated in water. Antigen retrieval was performed in a pressure cooker in EnVision FLEX Target Retrieval Solution, High pH (Dako) at 124°C, 15–16 psi for 4 min. Endogenous peroxidase activity was quenched with EnVision FLEX Peroxidase-Blocking Reagent (Dako) for 5 min at room temperature. bRWD1 or biotinyated isotype control IgG_1κ_ MAb was applied to the sections at 7 µg/mL and incubated at 4°C overnight. After washing, the VECTASTAIN ABC System (Vector Laboratories) was applied for 30 minutes at room temperature to detect biotin-labelled primary MAb. Freshly prepared 3,3′-diaminobenzidine (DAB; EnVision FLEX DAB+Chromogen in EnVision FLEX substrate buffer) was applied to the sections and incubated at room temperature until a suitable intensity of DAB staining had developed. The sections were counterstained with haematoxylin for 30 sec, blued in Scott's Tap Water for 1 min then dehydrated in ethanol for 1 minute (three times), cleared in xylene for 1 min (three times) and mounted in Pertex.

### Measurement of RWD1 affinity for RYK using ELISA and SPRi analysis

RYK binding was determined by ELISA as described previously [73] using immobilized RWD1 probed with purified hRYK.Fc. All SPRi analyses were performed at 25°C using HBST (10 mM HEPES, pH 7.4, 150 mM NaCl, 0.05% (v/v) Tween-20) as the running buffer. A ProteOn XPR36 SPRi biosensor, GLC sensor chips and coupling reagents (10 mM sodium acetate, pH 4.5; sulfo-N-hydroxysuccinimide (SNHS); 1-ethyl-3-(3-dimethylaminpropyl)-carbodiimide hydrochloride (EDC); and ethanolamine) were purchased from Bio-Rad Laboratories. All antibodies and ligands used for kinetic analysis were buffer-exchanged with PBS. Immobilizations were performed at 30 µL/min on the GLC chip. Prior to immobilization, chips were preconditioned with two sequential 10 sec pulses of 50 mM NaOH, 100 mM HCl and 0.5% SDS, followed by equilibration with HBST. For surface activation, 0.4 M EDC and 0.1 M SNHS were each diluted 50-fold in water and mixed together to give a final composition of 8 mM EDC in 2 mM SNHS. Separate vertical channels were activated with 150 µL of the EDC/SNHS mixture. MAbs for immobilization (RWD1 and human IgG (R&D Systems; control)) were diluted to 50 µg/mL in 10 mM acetate, pH 4.5, and coupled (3×150 µL) along separate vertical channels followed by an injection of ethanolamine (150 µL) to block the reaction spots. A second pulse of ethanolamine was injected in the horizontal direction. Final immobilization levels were 2046 RU for RWD1 and 1556 RU for control human IgG.

hRYKWD.Fc was injected (100 µL at a flow rate of 100 µL/min) in order of increasing concentration along each horizontal channel, allowing a full set of data for kinetic analysis to be obtained [56]. Dissociation was followed for a further 10 min. Following “one-shot kinetic analysis” [74], possible due to the sensor chip configuration of the ProteOn instrument, surfaces were regenerated using 0.85% phosphoric acid (30 µL) at a flow rate of 100 µL/min for repeat analyses.

All binding sensorgrams were collected, processed and analyzed using the integrated ProteOn Manager software (Bio-Rad Laboratories). Following a two-step background subtraction, using an interspot reference followed by the signals generated from the control antibody channel, resulting binding curves were fitted using the Langmuir model describing 1∶1 binding stoichiometry or with the Langmuir and mass transfer limitation model. Captured antibody interacting with the five concentrations of antigen was fitted globally to derive the ka, kd and KD.

### Neurite outgrowth assay

Embryos isolated from Swiss mice at embryonic day 15.5 were microdissected in chilled L15 medium (Life Technologies) and enzymatically dissociated in HBSS containing 0.05% trypsin and 0.1% DNase for 20 min at 37 °C. The resultant cell suspension was resuspended in serum-free medium consisting of a 1∶1 mixture of F12 and MEM supplemented with 15 mM HEPES buffer, 1 mM glutamine, 6 mg/mL glucose, 1.5 mg/mL bovine serum albumin and N2 supplement (all purchased from Life Technologies). Forty-eight–well plates were seeded at a density of 35,000 cells per well at 37 °C, 5% CO_2_.

After 72 h in culture, human IgG_1_ (Ancell Corporation; 50 µg/mL) or RWD1 antibody (50 µg/mL) was added to the cultures, and where appropriate, Wnt5a (R&D Systems; 300 ng/mL) was added 45 min thereafter. The cells remained in culture for a further 72 h before fixation with 4% paraformaldehyde and staining for βIII tubulin immunoreactivity (TUJ^+^) [38]. TUJ^+^ neurons were analyzed from four independent primary cultures. Under all culture conditions, sampling was commenced in the second field of view from the left-hand side of the culture well. The first 30 TUJ^+^ cells found to be measurable (neurites intact and distinguishable from other stained neurites, i.e. not intertwined with other TUJ^+^ neurites) were quantified in order to avoid any potential sampling bias. Photomicrographs of each neuron were taken using a 20× objective (Olympus IX71) and measurements of total neurite length obtained using Cell D software (Olympus). Groups were compared using a one-way ANOVA with Tukey's Multiple Comparison post-hoc test.

## Supporting Information

Table S1 Antibodies and scFv proteins reactive with RYK used in this study.
(DOCX)

Table S2 Fusion proteins used in this study and proteolytic fragments derived from them.
(DOCX)

Table S3 Details of modulators of proteolysis used in this study.
(DOCX)
