# Supplementary material for: A Fully Human Inhibitory Monoclonal Antibody to the Wnt Receptor RYK
Source: PLoS One. 2013 Sep 18;8(9):e75447. doi: 10.1371/journal.pone.0075447 (PMC3776778; doi:10.1371/journal.pone.0075447)
Supplement: Table S1 — Antibodies and scFv proteins reactive with RYK used in this study. (DOCX) [file pone.0075447.s001.docx]

**Table S1.** Antibodies and scFv proteins reactive with RYK used in this study.

| **Antibody or scFv** | **Antigen** | **Isotype** | **Host^a^** | **Epitope^b^** | **Reactive with** |
| --- | --- | --- | --- | --- | --- |
| Anti-Ryk^IC^ | GST.mRykIC | polyclonal | Rabbit | RykIC | Human, mouse |
| Anti-RYK^EC^ | hRYK.Fc |  |  | RykEC |  |
| RYK1, 2 & 3 | H-RYK-FLAG | IgM | Mouse | Human: RTIYD^216^ |  |
| 1B4 |  | IgG |  |  |  |
| 1G8 |  |  |  |  |  |
| 5E3 |  |  |  |  |  |
| 6G1 |  |  |  |  |  |
| scFv3 | hRYKWD.Fc | N/A | Human naïve synthetic scFv phage display library | RYK WIF domain |  |
| scFvN3 |  |  |  |  |  |
| RWD1 (scFv3-derivative) |  | IgG_1κ_ (fully human) | HEK293T, CHO |  |  |
| 1. Organism specified when immunization performed; cell line specified when used for recombinant MAb production. 2. Peptide sequence specified where known; otherwise, the relevant region or domain is identified.   GST, glutathione-*S*-transferase, IC, intracellular region; EC, extracellular region; m, mouse; h or H, human. | | | | | |
