# Supplementary material for: A Fully Human Inhibitory Monoclonal Antibody to the Wnt Receptor RYK
Source: PLoS One. 2013 Sep 18;8(9):e75447. doi: 10.1371/journal.pone.0075447 (PMC3776778; doi:10.1371/journal.pone.0075447)
Supplement: Table S2 — Fusion proteins used in this study and proteolytic fragments derived from them. (DOCX) [file pone.0075447.s002.docx]

**Table S2.** Fusion proteins used in this study and proteolytic fragments derived from them.

| **Name** | **Type** | **Description** | **Utility** |
| --- | --- | --- | --- |
| H-RYK-FLAG | Fusion | Entire human RYK EC region with C-terminal FLAG epitope tag | Antigen for generating MAbs |
| GST.mRykIC |  | C-terminal fusion of GST to the entire mouse Ryk IC region | Antigen for generating pAbs |
| hRYK.Fc |  | C-terminal fusion of entire human RYK EC region with the hinge and Fc regions of human IgG_1_ and a C-terminal FLAG epitope tag | Ligand trap; epitope mapping |
| hRYKWD.Fc |  | C-terminal fusion of human RYK WIF domain with the hinge and Fc regions of human IgG_1_ and a C-terminal FLAG epitope tag | Ligand trap; epitope mapping; antigen for generating MAbs |
| Ryk.Fc |  | C-terminal fusion of entire mouse Ryk EC region with the hinge and Fc regions of human IgG_1_ and a C-terminal FLAG epitope tag | Ligand trap; epitope mapping; ligand-binding studies |
| RykΔWD.Fc |  | C-terminal fusion of mouse Ryk EC region (WIF domain deleted) with the hinge and Fc regions of human IgG_1_ and a C-terminal FLAG epitope tag | Epitope mapping; ligand-binding studies |
| RykWD.Fc |  | C-terminal fusion of mouse Ryk WIF domain with the hinge and Fc regions of human IgG_1_ and a C-terminal FLAG epitope tag | Ligand trap; epitope mapping; ligand-binding studies |
| RykΔN.Fc |  | C-terminal fusion of mouse Ryk EC region (peptide N-terminal to the WIF domain deleted) with the hinge and Fc regions of human IgG_1_ and a C-terminal FLAG epitope tag | Ligand trap; epitope mapping |
| RykΔC.Fc |  | C-terminal fusion of mouse Ryk EC region (EC juxtamembrane region deleted) with the hinge and Fc regions of human IgG_1_ and a C-terminal FLAG epitope tag | Ligand trap; epitope mapping; ligand-binding studies |
| hRYKFCT |  | Full-length human RYK with the C-terminus **DYKDDDDK**HAALGAYV (FLAG epitope bolded) | Positive control for flow cytometry |
| m(h)M2RFCT |  | Full-length mouse Ryk (or human RYK) with two N-terminal Myc epitope tags and the C-terminus **DYKDDDDK**HAALGAYV (FLAG epitope bolded) | Analysis of Ryk proteolytic cleavage events |
| mM2RFCT.CM |  | Compound mutant of mM2RFCT encoding KRRK176;KK181;K186 to QQQQ176;QQ181;Q186 |  |
| mM2RFCT.TB |  | Tetrabasic mutant of mM2RFCT encoding KRRK176 to QQQQ176 |  |
| mM2RFCT.DB |  | Dibasic mutant of mM2RFCT encoding  KK181 to QQ181 |  |
| mM2RFCT.MB |  | Monobasic mutant of mM2RFCT encoding K186Q |  |
| mM2RFCT.V594A |  | Mutant of mM2RFCT encoding V594A at extreme C-terminus |  |
| c-Met.FLAG |  | Mouse c-Met with a C-terminal FLAG epitope tag | Positive control substrate for furin activity |
| p190 c-Met |  | Full-length uncleaved mouse c-Met with C-terminal FLAG epitope tag |  |
| α_1_-PDX.FLAG |  | α_1_-Protease inhibitor Portland with a C-terminal FLAG epitope tag | Highly specific inhibitor of furin activity |
| Ryk-FL |  | Full-length transmembrane Ryk | N/A |
| Wnt1 |  | Mouse Wnts with five tandem C-terminal Myc epitope tags | Ligands for the Ryk WIF domain |
| Wnt3a |  |  |  |
| Wnt5a |  |  |  |
| hM2R^WIF1-WD^FCT |  | Human M2RFCT with the RYK WIF domain replaced by the Wnt-binding WIF domain from WIF1 | Controls for binding specificity of RWD1 |
| hM2R^ROR2-CRD^FCT |  | Human M2RFCT with the RYK WIF domain replaced by the Wnt-binding cysteine-rich domain (CRD) from ROR2 |  |
| p140 c-Metβ | Fragment | C-terminal transmembrane furin cleavage product of p190 c-Met with C-terminal FLAG epitope tag | Positive control product of furin activity |
| Ryk-NTF |  | N-terminal metalloprotease cleavage fragment of Ryk | N/A |
| Ryk-CTF |  | C-terminal metalloprotease cleavage fragment of Ryk with transmembrane helix |  |
| Rβ |  | Predicted internal peptide fragment produced by γ-secretase–mediated cleavage of Ryk-CTF, analogous to the Aβ peptide |  |
| Ryk-ICD |  | C-terminal cleavage fragment produced by γ-secretase–mediated cleavage of Ryk-CTF (cytoplasmic, soluble) |  |
| GST, glutathione-*S*-transferase, IC, intracellular region; EC, extracellular region; m, mouse; h or H, human; WIF, Wnt-inhibitory factor; CTF, carboxyl-terminal fragment; NTF, amino-terminal fragment; ICD, intracellular domain (region). | | | |
