# Supplementary material for: A Fully Human Inhibitory Monoclonal Antibody to the Wnt Receptor RYK
Source: PLoS One. 2013 Sep 18;8(9):e75447. doi: 10.1371/journal.pone.0075447 (PMC3776778; doi:10.1371/journal.pone.0075447)
Supplement: Table S3 — Details of modulators of proteolysis used in this study. (DOCX) [file pone.0075447.s003.docx]

**Table S3.** Details of modulators of proteolysis used in this study.

| **Agent** | **Full name or composition** | **Relevant activity** | **Final concentration** |
| --- | --- | --- | --- |
| α_1_-PDX.FLAG | α_1_-Antitrypsin Portland with a C-terminal FLAG epitope tag | Highly potent inhibitor of furin (and to a lesser extent PC5 and PACE4) | Transiently transfected |
| decanoyl-RVKR-cmk | Decanoyl-arginyl-valyl-lysyl-arginyl-chloromethylketone | Blocks activity of basic-specific proprotein convertases: PC1, PC2, furin, PC4, PC5, PACE4, and PC7 | 50 μM |
| PMA | Phorbol-12-myristate-13-acetate | Mimic of triacylglycerol, activator of PKC | 1 μM |
| TFP | Trifluoroperazine; 10-[3-(4-methylpiperazin-1-yl)propyl]-  2-(trifluoromethyl)-10H-phenothiazine | Calmodulin inhibitor | 100 μM |
| Suramin | Germanin; 309 F; 309 Fourneau; Bayer 205; Moranyl; Naganin; Naganine | Releases Wnts from the extracellular matrix | 1 mM |
| fMLF | N-formylmethionyl-leucyl-phenylalanine | An activator of receptor shedding in some contexts | 100 μM |
| A23187 | Calcimycin | Divalent cation ionophore | 1 μM |
| PBSS | Ca^2+^/Mg^2+^-supplemented phosphate-buffered saline | None; vehicle for pervanadate |  |
| Pervanadate | 100 μM Na_3_VO_4_, 200 μM H_2_O_2_ in PBSS | Potent protein phosphotyrosyl phosphatase inhibitor | 100 μM Na_3_VO_4_, 200 μM H_2_O_2_ in PBSS |
| Actinonin | (2R)-N4-hydroxy-N1-{(2S)-1-[(2S)-2-(hydroxymethyl)pyrrolidin-1-yl]-3-methyl-1-oxobutan-2-yl}-2-pentylbutanediamide | Leucine aminopeptidase inhibitor | 50 μM |
| TAPI-0 | N-(R)-[2-(hydroxyaminocarbonyl)methyl]-4-methylpentanoyl-L-naphthylalanyl-L-alanine amide; TNF-α Protease Inhibitor-0 | Hydroxamate-based inhibitors of metalloproteases | 100 μM |
| TAPI-1 | N-(R)-[2-(hydroxyaminocarbonyl)methyl]-4-methylpentanoyl-L-naphthylalanyl-L-alanine-2-aminoethyl amide; TNF-α Protease Inhibitor-1 |  |  |
| TAPI-2 | N-(R)-[2-(hydroxyaminocarbonyl)methyl]-4-methylpentanoyl-L-t-butyl-alanyl-L-alanine-2-aminoethyl amide; TNF-α Protease Inhibitor-2 |  |  |
| Phosphoramidon | N-(α-rhamno­pyranosyl­phos­phono)-L-leucyl-L-tryptophan | Inhibits thermolysin and other metallo-endopeptidases, plus enkephalinase | 50 μM |
| GM6001Ө | N-t-butoxycarbonyl-L-leucyl-L-tryptophan methylamide | Inactive analog of GM6001 |  |
| GM6001 | Ilomastat; N-[(2R)-2-(hydroxamidocarbonylmethyl)-4-methylpentanoyl]-L-tryptophan methylamide | MMP and collagenase inhibitor |  |
| SB203580 | 4-(4-fluorophenyl)-2-(4-methylsulfinylphenyl)-5-(4-pyridyl)-imidazole | Selective inhibitor of p38 MAPK | 10 μM |
| U0126 | 1,4-Diamino-2,3-dicyano-1,4-bis(2-aminophenylthio)-butadiene | Inhibitor of MEK1 and MEK2 | 5 μM |
| PC, proprotein convertase; PKC, protein kinase C; MMP, matrix-type metalloprotease; TACE, TNF-α converting enzyme; MAPK, mitogen-activated protein kinase; TAPI, TNF-α protease inhibitor. | | | |
